# Minimally invasive resection of solid intraventricular lesions via single burr-hole ventriculoscopy

**DOI:** 10.3389/fneur.2026.1864946

**Published:** 2026-06-15

**Authors:** Xinghua Xu, Jiashu Zhang, Zhichao Gan, Qun Wang, Haoyang Zheng, Shiyu Zhang, Xiaolei Chen

**Affiliations:** 1 Department of Neurosurgery, The First Medical Center of Chinese PLA General Hospital, Beijing, China; 2 Medical School of Chinese PLA, Beijing, China

**Keywords:** burr-hole surgery, intraventricular lesion, minimally invasive surgery, neurosurgical oncology, ventriculoscopy

## Abstract

**Background:**

Maximal safe resection of intraventricular lesions remains challenging. This study evaluated the feasibility, technical workflow, and clinical outcomes of single burr-hole pure ventriculoscopic resection for small solid intraventricular lesions.

**Methods:**

We retrospectively reviewed patients who underwent single burr-hole pure ventriculoscopic resection of solid intraventricular lesions. Clinical, radiological, operative, and postoperative data were collected. All patients received high-resolution 3D-SPACE MRI, and virtual ventriculoscopic reconstruction using 3D Slicer software for individualized trajectory planning. Lesions were resected using a 6-mm rigid ventriculoscope. Postoperative MRI was performed to evaluate the extent of resection, and patients were followed for neurological outcomes and complications.

**Results:**

Thirty-eight patients (median lesion diameter: 17 mm) underwent surgery. Gross-total resection was achieved in 92.1% (35/38) of cases. No seizures, cerebrospinal fluid leakage, permanent neurological deficits, or mortality occurred. Complications included transient diplopia in 2 patients (5.3%) and one intracranial infection (2.6%). Pathological diagnoses included cavernous malformations in 14 patients, subependymomas in 10, meningiomas in 8, low-grade gliomas in 3, and neurofibroma, pseudoaneurysm, and inflammatory granuloma in 1 patient each. During a mean follow-up of 60 months, no definite lesion recurrence was observed, and one patient developed delayed hydrocephalus that was successfully treated endoscopically. The planned surgical trajectories showed good concordance with intraoperative findings, supporting the accuracy of preoperative virtual planning.

**Conclusion:**

Single burr-hole pure ventriculoscopic resection is a safe and feasible minimally invasive approach for selected small intraventricular lesions, enabling high rates of complete resection with low morbidity and favorable long-term outcomes.

## Introduction

Intraventricular lesions represent a distinct and technically demanding category of intracranial pathology. Due to their deep-seated location within the ventricular system and close proximity to critical periventricular structures—including the fornix, thalamus, internal capsule, and deep venous system—surgical access and safe resection remain highly challenging (1, 2). Even minimal operative manipulation in these regions may result in significant neurological deficits, such as memory impairment, hemiparesis, or visual field loss (3). Traditional microsurgical approaches, including transcallosal and transcortical craniotomies, require substantial cortical or callosal dissection to access deep ventricular lesions. These approaches are associated with notable risks, including seizures, disconnection syndromes, hydrocephalus, and cerebral edema (4). Furthermore, the narrow operative corridor and limited visualization of deep structures substantially increase surgical complexity and perioperative risk.

Ventriculoscopic techniques have emerged as a minimally invasive alternative for selected intraventricular pathologies (5–7). Advances in high-definition optics, angled visualization, and specialized instruments enable access to deep ventricular regions through minimally invasive cranial corridors while maintaining a panoramic surgical view (8, 9). However, most existing ventriculoscopic applications have primarily focused on cystic or obstructive lesions, and the resection of solid intraventricular lesions remains technically challenging, particularly with respect to visualization, hemostasis, and controlled resection.

Single burr-hole ventriculoscopy has the potential to further minimize surgical invasiveness by reducing cortical disruption and enabling individualized trajectory planning through a single, precisely defined entry point. This approach may facilitate effective lesion access while preserving surrounding neural structures (10, 11). Nevertheless, evidence regarding its safety, feasibility, and clinical efficacy for solid intraventricular lesions remains limited. In this study, we evaluated the clinical applicability of single burr-hole pure ventriculoscopic resection for solid intraventricular lesions, with particular emphasis on the extent of resection and perioperative morbidity.

## Materials and methods

### Patient population

We retrospectively reviewed patients who underwent single burr-hole pure ventriculoscopic resection of solid intraventricular lesions in our department between January 2013 and June 2025. An approximately 8-mm burr hole was used as the surgical entry point. Clinical data were collected from medical records, including demographic characteristics, presenting symptoms, imaging findings, lesion location and size, pathological diagnosis, operative details, and postoperative complications. Patients were included if they had radiologically confirmed solid intraventricular lesions and underwent resection using a single burr-hole pure ventriculoscopic approach. Patient selection was primarily based on lesion size, lesion location within the ventricular system, relationship to critical neurovascular structures, ventricular anatomy, and the feasibility of achieving adequate visualization and safe surgical manipulation through a single burr-hole corridor. Cases with large, highly vascular lesions, extensive calcification, dense adhesions, marked mass effect requiring wider exposure, or anticipated difficulty in hemostasis were generally considered unsuitable for this approach.

### Virtual ventriculoscopy

In addition to conventional MRI sequences, including T1- and T2-weighted imaging, all patients underwent three-dimensional sampling perfection with application-optimized contrasts using different flip angle evolutions (3D-SPACE) imaging to obtain high-resolution volumetric data of the ventricular system. The 3D-SPACE sequence was acquired with a repetition time (TR) of 1500 ms, an echo time (TE) of 227 ms, a matrix size of 640 × 640, a field of view (FOV) of 225 × 225 mm, and a slice thickness of 0.625 mm, allowing detailed delineation of cerebrospinal fluid (CSF) spaces and ventricular anatomy. The DICOM data were imported into 3D Slicer (version 5.8; https://www.slicer.org) for virtual ventriculoscopic reconstruction. Image preprocessing included isotropic resampling and intensity normalization. Semi-automatic segmentation of the ventricular system and lesions was performed using the “Segment Editor” module with thresholding and manual refinement (12). A virtual endoscopic simulation was then generated by navigating within the reconstructed ventricular cavity, allowing detailed assessment of lesion morphology, spatial relationships with critical structures, and potential surgical corridors. The preoperative planning results were used to guide the intraoperative approach, facilitating precise and safe ventriculoscopic resection.

### Operative technique

Surgical approaches were selected according to lesion location and individualized preoperative trajectory planning. Frontal transcortical approaches were primarily used for lesions involving the frontal horn, body of the lateral ventricle, foramen of Monro, third ventricle, or cerebral aqueduct, whereas parietal transcortical approaches were used for lesions located in the atrium (trigone) or temporal horn. In all cases, the entry point and surgical trajectory were individually determined based on preoperative virtual planning. After standard preparation and draping, an approximately 8-mm burr hole was created using a high-speed drill. Following dural opening, a rigid ventriculoscope (outer diameter 6 mm; Karl Storz, Tuttlingen, Germany) was introduced into the ventricle along the predefined trajectory under neuronavigation guidance. The surgical corridor was established while minimizing cortical disruption, and the lesion was identified and exposed under endoscopic visualization. Flexible electronic endoscope was used in cases with lesions located near the cerebral aqueduct to obtain a better observation angle and assess the extent of lesion resection.

**Figure 1:**
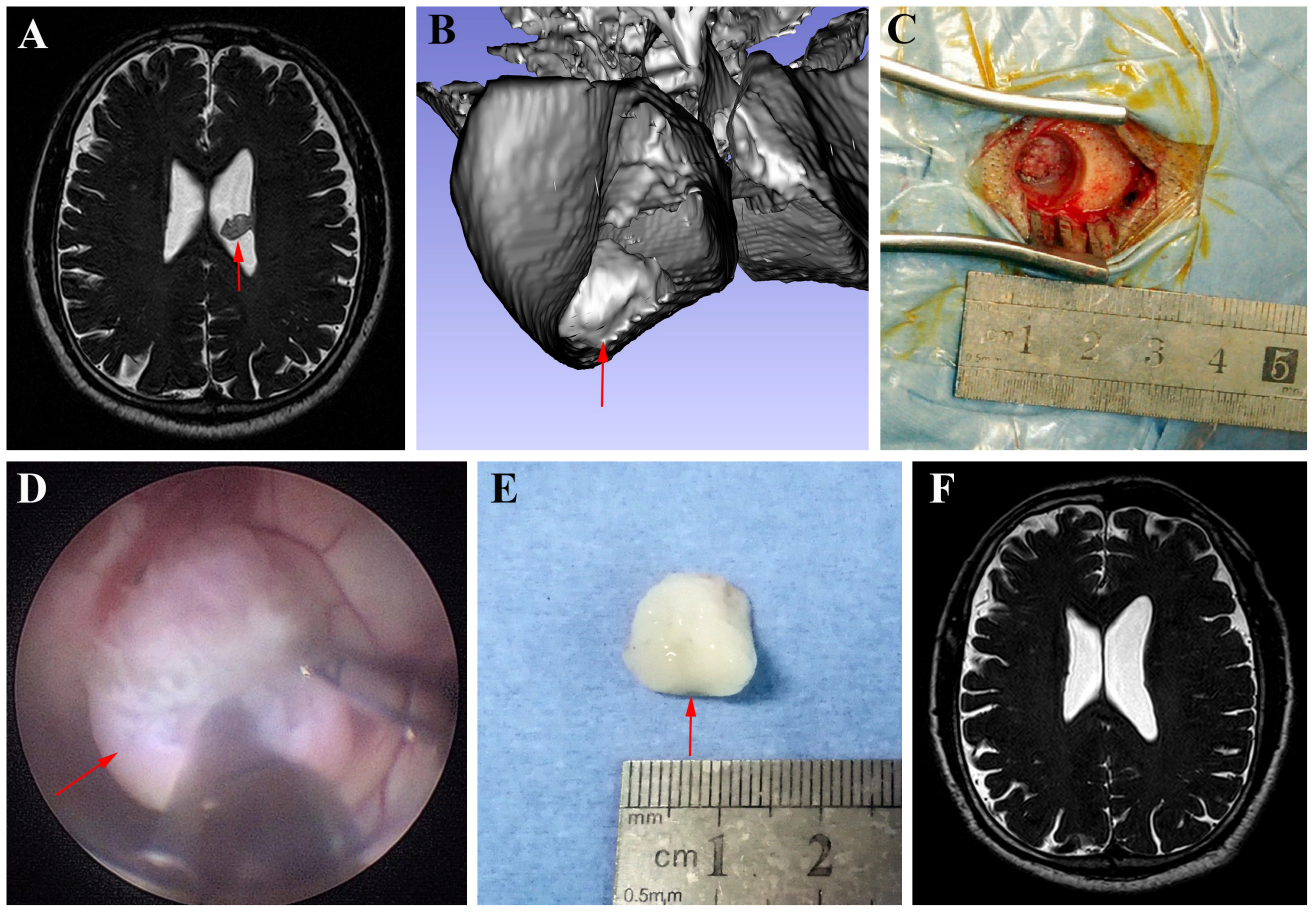
Single burr-hole ventriculoscopic resection of a lateral ventricular lesion. **(A)** Preoperative 3D-SPACE MRI. **(B)** Virtual ventriculoscopic simulation. **(C)** Skin incision and burr hole. **(D)** Intraoperative endoscopic view demonstrating lesion resection. **(E)** Resected lesion specimen. **(F)** Postoperative 3D-SPACE MRI. Red arrows denote the lesion.

Lesion resection was performed in a piecemeal fashion using standard ventriculoscopic instruments, including bipolar coagulation, lesion forceps, endoscopic scissors, and a neodymium-doped yttrium aluminum garnet (Nd:YAG) contact laser (Ligenesis-MY100C; Radium Health Science and Technology; wavelength 1064 nm) (13). These instruments facilitated controlled dissection, coagulation, and debulking within the confined endoscopic workspace. Larger lesion fragments were removed by en bloc withdrawal of the forceps and the endoscopic sheath to ensure safe extraction. Hemostasis was achieved using bipolar coagulation for active bleeding and continuous warm saline irrigation for minor venous oozing. For lesions involving the third ventricle or causing obstruction at the foramen of Monro, endoscopic third ventriculostomy (ETV) was performed when indicated to prevent or treat obstructive hydrocephalus. After confirmation of satisfactory resection and hemostasis, the endoscope was withdrawn. Oxidized regenerated cellulose was applied when necessary, followed by standard layered closure. The surgical schematic is shown in Figure 1.

### Follow-up

All patients underwent postoperative MRI scan using the same sequences as in the preoperative evaluation. Gross total resection (GTR) was defined as the absence of any visible residual lesion on postoperative MRI, whereas subtotal resection (STR) was defined as the presence of residual lesion. Neurological status and postoperative complications were assessed through clinical examinations in the immediate postoperative period and during follow-up. Follow-up evaluations were conducted via outpatient visits or telephone interviews, supplemented by radiological assessments when available. All patients underwent neurological functional assessment using the modified Rankin Scale (mRS). Preservation of the preoperative neurological status with an mRS score of 0–1 at 30-day follow-up was defined as no permanent neurological deficits.

### Statistical analysis

Statistical analysis was primarily descriptive. Continuous variables were presented as mean ± standard deviation or median (range), while categorical variables were summarized as frequencies and percentages. Statistical analyses were performed using SPSS software (version 27). Given the descriptive design of the study and the absence of a control group, no formal comparative statistical analyses were performed.

## Results

### Patient characteristics and surgical outcomes

Between January 2013 and June 2025, a total of 38 patients (23 females, 15 males) underwent single burr-hole pure ventriculoscopic resection of solid intraventricular lesions at our institution. Patient age ranged from 1 to 63 years (median, 29 years). Lesion diameter ranged from 9 to 33 mm, with a median size of 17 mm. Regarding lesion location, 21 lesions (55.3%) were located in the lateral ventricle, 8 (21.1%) in the third ventricle, 5 (13.2%) at the foramen of Monro, and 4 (10.5%) adjacent to the cerebral aqueduct. Common presenting symptoms included headache (24 patients, 63.2%), confusion (18, 47.4%), and gait ataxia (13, 34.2%). Four patients (10.5%) presented with impaired consciousness (lethargy or stupor), whereas 8 patients (21.1%) were asymptomatic, with lesions detected incidentally on imaging. Obstructive hydrocephalus was present in 5 patients (13.2%) at presentation. Most lesions were small and located in deep ventricular regions. Detailed patient characteristics, lesion distribution, and surgical outcomes were summarized in Table 1.

**Table 1:** Patient demographics and clinical characteristics (*n* = 38).

Parameter	Value
Age	
Median (range)	29 (1–63)
Sex	
Female	23 (60.5%)
Male	15 (39.5%)
Location	
Lateral ventricle	21 (55.3%)
Third ventricle	8 (21.1%)
Foramen of Monro	5 (13.2%)
Cerebral aqueduct	4 (10.5%)
Clinical manifestations	
Headache	24 (63.2%)
Confusion	18 (47.4%)
Gait ataxia	13 (34.2%)
Lethargy or stupor	4 (10.5%)
Diplopia	2 (5.3%)
Asymptomatic	8 (21.1%)
Lesion diameter (mm)	
Median (range)	17 (9–33)
Extent of resection	
Gross-total resection	35 (92.1%)
Subtotal resection	3 (7.9%)
Surgical complications	
Transient diplopia	2 (5.3%)
Intracranial infection	1 (2.6%)
Pathological findings	
Cavernous malformation	14 (36.8%)
Subependymoma	10 (26.3%)
Meningioma	8 (21.1%)
Low-grade glioma	3 (7.9%)
Neurofibroma	1 (2.6%)
Pseudoaneurysm	1 (2.6%)
Inflammatory granuloma	1 (2.6%)
Follow-up, months	
Mean ± SD	60.2 ± 32.5
Range	5–148

Radiographically confirmed GTR was achieved in 35 of 38 patients (92.1%), whereas STR was observed in 3 patients (7.9%) based on postoperative MRI findings. The median operative time was 3.2 h (range, 2.1–4.8 h), and the estimated mean intraoperative blood loss was 45 mL. No patient required intraoperative blood transfusion or conversion to craniotomy during the procedure. No patient required reoperation for postoperative hemorrhage. No seizures, CSF leakage, new permanent neurological deficits, or perioperative mortality were observed. Transient diplopia occurred in two patients (5.3%), and one patient (2.6%) developed a postoperative intracranial infection. The patient with intracranial infection developed fever (>38 °C) lasting 3 days with a CSF white blood cell count >300 × 10^6^/L. Although CSF culture was negative, the patient recovered completely after 1 week of lumbar cistern drainage and intravenous antibiotic therapy. Histopathological analysis revealed cavernous malformations in 14 patients (36.8%), subependymomas in 10 (26.3%), meningiomas in 8 (21.1%), low-grade gliomas in 3 (7.9%), and one case each (2.6%) of neurofibroma, pseudoaneurysm (preoperatively diagnosed as cavernous malformation), and inflammatory granuloma.

The mean follow-up duration was 60.2 ± 32.5 months (range, 5–148 months). Overall, no definite lesion recurrence or progression was observed during follow-up. One patient developed delayed hydrocephalus 2 years after the initial procedure. High-resolution 3D-SPACE MRI demonstrated restenosis of the ETV stoma, and repeat ETV was successfully performed. The patient remained free of recurrent hydrocephalus during an additional 5 years of follow-up. Among the three patients who underwent STR, postoperative MRI surveillance was performed at 1 month, 3 months, and at 6-month intervals thereafter. No radiological evidence of lesion progression or recurrence was observed during follow-up periods of 2.5 years, 3 years, and 4 years 10 months, respectively.

### Illustrative case

A 42-year-old man presented with a 3-month history of photophobia and diplopia. Ten years earlier, he had been diagnosed with a midbrain cavernous malformation and had undergone Gamma Knife radiosurgery with incomplete symptom control. T2-weighted MRI demonstrated a heterogeneous lesion in the dorsal midbrain with a characteristic mixed-signal core surrounded by a hypointense hemosiderin rim. On 3D-SPACE MRI, the lesion was clearly visualized at the level of the cerebral aqueduct, causing obstruction with obliteration of the normal CSF flow void. Preoperative virtual ventriculoscopy facilitated individualized trajectory planning and confirmed endoscopic accessibility of the lesion. The lesion was diagnosed as cavernous malformation involving the cerebral aqueduct, and the patient underwent endoscopic resection via a single frontal burr-hole approach.

**Figure 2:**
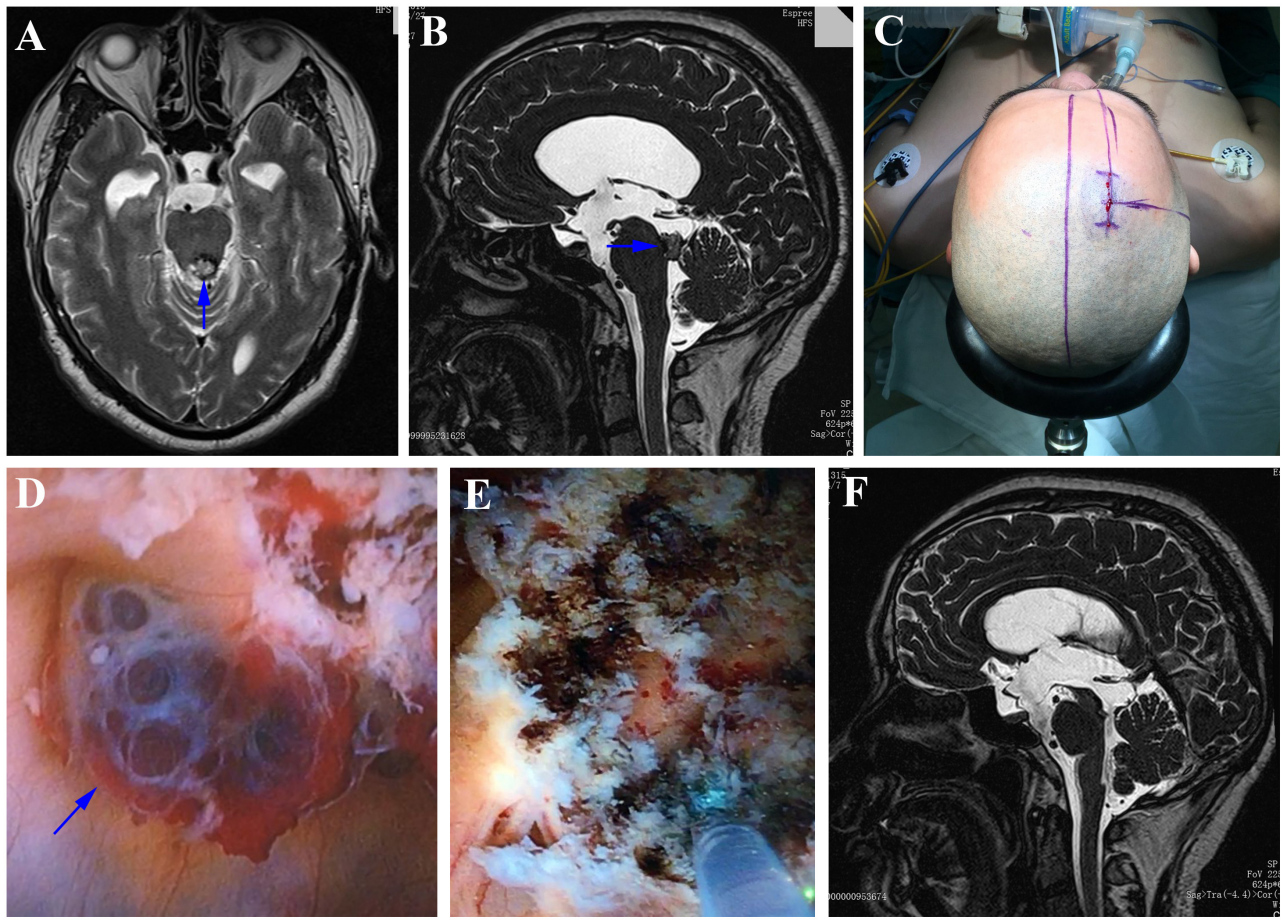
Illustrative case of single burr-hole ventriculoscopic resection. **(A)** Preoperative T2-weighted MRI demonstrating a posterior midbrain lesion. **(B)** Sagittal 3D-SPACE MRI showing aqueductal obstruction by the lesion. **(C)** Patient positioning and surgical incision. **(D)** Intraoperative endoscopic view of the lesion. **(E)** Laser-assisted lesion resection. **(F)** Postoperative 3D-SPACE MRI. Blue arrows denote the lesion.

Intraoperatively, a flexible electronic endoscope was additionally used to obtain improved visualization of the aqueductal region, whereas all surgical procedures were performed under a rigid ventriculoscope. A nodular lesion with well-defined margins was identified, completely obstructing the aqueduct. The lesion was first coagulated to reduce vascularity, followed by progressive debulking and complete excision using endoscopic instruments and a contact laser. Subsequently, aqueductoplasty and exploration of the fourth ventricle were performed. ETV was additionally performed to reduce the risk of postoperative obstructive hydrocephalus. Postoperative MRI confirmed complete resection, and histopathological examination verified the diagnosis of cavernous malformation (Figure 2). The patient experienced resolution of photophobia and marked improvement of diplopia, with no new neurological deficits. He was discharged on postoperative day 7 and remained free of recurrence and hydrocephalus during 5 years of follow-up period.

## Discussion

Intraventricular lesions remain among the most technically challenging entities in neurosurgery due to their deep anatomical location, proximity to critical structures, and the difficulty of achieving adequate exposure without cortical or white matter transgression (14). Conventional craniotomy typically requires a 5–6 cm craniotomy and extensive cortical access, which is associated with an increased risk of retraction injury, venous disruption, and postoperative neurological morbidity (15–17). These limitations have driven the development of minimally invasive approaches aimed at reducing surgical trauma while preserving efficacy. Ventriculoscopy, with its panoramic visualization, continuous irrigation, and improved illumination, has emerged as a valuable tool for ventricular surgery (18, 19). However, even small-bone-flap endoscope-assisted approaches (≈2 cm) still require cortical entry and may be constrained by fixed working channels (20–22).

In this context, we developed a single burr-hole pure ventriculoscopic technique for the resection of intraventricular lesions, representing an additional step in the evolution of minimally invasive ventricular surgery. In this consecutive series of 38 patients, a high rate of GTR (92.1%) was achieved with a favorable safety profile. No cases required reoperation for postoperative hemorrhage, and no seizures, CSF leakage, or perioperative mortality were observed. Long-term follow-up demonstrated favorable outcomes, with no recurrence after GTR and stable residual lesions in patients undergoing STR. One patient developed delayed hydrocephalus, which was successfully managed endoscopically.

To further contextualize these findings, a summary table (Table 2) was added to systematically compare recently published studies with similar surgical approaches, including endoscope-assisted microsurgery and tubular retractor-assisted techniques (1, 2, 15, 23, 24). Key outcome measures, such as gross-total resection rate and perioperative complication rate, were extracted and compared with those of the present series, providing a clearer benchmark for evaluating the efficacy and safety of the single burr-hole pure ventriculoscopic approach. Several technical factors may have contributed to these outcomes. The use of a small burr hole minimizes cortical disruption and may reduce the risk of venous injury while allowing flexible trajectory planning tailored to lesion location. High-definition endoscopic visualization provides enhanced illumination and a panoramic visualization of the ventricular system, facilitating dissection and hemostasis. In addition, angled and flexible endoscopes may improve access to deep or obliquely oriented lesions (25).

**Table 2:** Comparison of surgical outcomes across different surgical approaches.

Study	Surgical approach	Lesion type	GTR rate (%)	Complications
D'Angelo et al. (15)	Conventional craniotomy	Lateral ventricle tumors	82% (59/72)	11% (8/72)
Charalampaki et al. (1)	Endoscope-assisted microneurosurgery	Lateral and third ventricle tumors	78.5% (28/35)	3 parinaud syndrome and 1 motor weakness
Kassam et al. (2)	Endoscope-assisted microneurosurgery	Subcortical brain lesions	66.7% (14/21)	1 infection and 1 pulmonary embolus
Boogaarts et al. (24)	Neuroendoscopy	Third ventricular colloid cysts	57.5% (46/80)	28.9% (26/90)
Wilson et al. (23)	Neuroendoscopy	Third ventricular colloid cysts	95% (21/22)	1 subdural hematoma
This study	Single burr-hole ventriculoscopy	Solid intraventricular lesions	92.1% (35/38)	2 transient diplopia and 1 intracranial infection

This evolution from traditional craniotomy to small-bone-flap endoscopic approaches and ultimately to single burr-hole pure ventriculoscopy reflects ongoing efforts to minimize surgical trauma while maintaining efficacy. The present results suggest that, in appropriately selected patients, this approach can achieve satisfactory resection with a favorable safety profile. Compared with conventional craniotomy, it may reduce cortical disruption and approach-related morbidity. However, these potential advantages should be interpreted with caution given the retrospective design and limited sample size.

Nonetheless, several limitations should be acknowledged. The narrow working corridor restricts instrument maneuverability, and achieving hemostasis may be more challenging than in open microsurgical procedures. In addition, the technique requires substantial experience in ventriculoscopy and may involve a significant learning curve, particularly over a long study period. This was also a retrospective single-center study with a relatively small sample size, which may introduce selection bias and limit generalizability. Furthermore, this minimally invasive approach was applied only in highly selected patients based on lesion size, ventricular anatomy, location, vascularity, and surgical accessibility. Finally, the absence of a control group limits direct comparison with conventional microsurgical or other minimally invasive approaches.

In summary, single burr-hole pure ventriculoscopic resection represents a minimally invasive approach for selected intraventricular lesions, enabling effective lesion removal with minimal cortical disruption and favorable clinical outcomes. Further prospective, multicenter studies are warranted to validate its broader applicability.

## Data Availability

The raw data supporting the conclusions of this article will be made available by the authors, without undue reservation.
